# Self-Reported Increased Confusion or Memory Loss and Associated Functional Difficulties Among Adults Aged ≥60 Years — 21 States, 2011

**Published:** 2013-05-10

**Authors:** Mary L. Adams, Angela J. Deokar, Lynda A. Anderson, Valerie J. Edwards

**Affiliations:** On Target Health Data LLC, West Suffield, Connecticut; Div of Population Health, National Center for Chronic Disease Prevention and Health Promotion, CDC

Declines in cognitive function vary among persons and can include changes in attention, memory, learning, executive function, and language capabilities that negatively affect quality of life, personal relationships, and the capacity for making informed decisions about health care and other matters (1). Memory problems typically are one of the first warning signs of cognitive decline, and mild cognitive impairment might be present when memory problems are greater than normal for a person’s age but not as severe as problems experienced with Alzheimer’s disease (2,3). Some, but not all, persons with mild cognitive impairment develop Alzheimer’s disease; others can recover from mild cognitive impairment if certain causes (e.g., medication side effects or depression) are detected and treated (3). In 2012, the U.S. Department of Health and Human Services published the *National Plan to Address Alzheimer’s Disease,* calling for expanding data collection and surveillance efforts to track the prevalence and impact of Alzheimer’s and other types of dementia (4). To estimate the prevalence of self-reported increased confusion or memory loss and associated functional difficulties among adults aged ≥60 years, CDC analyzed data from 21 states that administered an optional module in the 2011 Behavioral Risk Factor Surveillance System (BRFSS) survey. The results indicated that 12.7% of respondents reported increased confusion or memory loss in the preceding 12 months. Among those reporting increased confusion or memory loss, 35.2% reported experiencing functional difficulties. These results provide baseline information about the number of noninstitutionalized older adults with increased confusion or memory loss that is causing functional difficulties and might require services and supports now or in the future.

BRFSS consists of annual state-based telephone surveys of randomly selected noninstitutionalized U.S. adults aged ≥18 years regarding health practices and risk behaviors linked to chronic diseases, injuries, and preventable infectious diseases.* In 2011, all 50 states and the District of Columbia conducted the BRFSS survey by landline and cellular telephones, and the median survey response rate was 49.7%. In 2011, 21 states† included a 10-question optional cognitive impairment module§ in their BRFSS surveys. Because only seven of the 21 states conducted cell phone interviews in addition to landline telephone interviews, this analysis was restricted to landline respondents aged ≥60 years from the 21 states.¶ The median landline response rate among the 21 states was 53.4%, and the rates ranged from 37.4% in California to 66.0% in Nebraska.** This analysis was further limited to the 59,852 adults aged ≥60 years with nonmissing responses to the first question in the module.

Respondents who answered affirmatively to the question, “During the past 12 months, have you experienced confusion or memory loss that is happening more often or is getting worse?” were categorized as reporting increased confusion or memory loss. Functional difficulties were identified among these persons if they responded, “always,” “usually,” or “sometimes” to one of two questions about whether confusion or memory loss interfered with their “ability to work, volunteer, or engage in social activities,” or caused them to “give up household activities or chores” that they “used to do.” Additional questions addressed the need for assistance, getting care or assistance from a family member or friend, and discussing increased confusion or memory loss with a health-care provider. Respondents who declined to answer, had a missing answer, or who answered “don’t know/not sure” were excluded from the analyses involving those variables.

Respondents were categorized by age group, sex, race/ethnicity,†† education level, disability status,§§ veteran status, and employment status. BRFSS landline weights were used to adjust for the probability of selection and to reflect the total adult population in each state by age group, race/ethnicity, education level, marital status, and home ownership status. To account for the complex sampling design, weighted data were analyzed using statistical software.

In 2011, 12.7% of respondents reported increased confusion or memory loss during the preceding 12 months, and 35.2% of those persons reported functional difficulties (Table 1). The percentage reporting confusion or memory loss was significantly higher among the following: persons aged ≥85 years (15.6%) compared with those aged 60–64 years (12.0%) and 65–74 years (11.9%); Hispanics or Latinos (16.9%) compared with whites (12.1%); persons with less than a high school education (16.2%) compared with persons with more education; persons who reported they were disabled (20.2%) compared with persons who were not disabled (7.5%); and persons who were unable to work (28.3%) compared with those who were employed (7.8%), unemployed (16.4%), homemakers (11.8%), students (3.9%), and retirees (12.3%) (Table 1).

Among those reporting increased confusion or memory loss, significant differences in the percentage with functional difficulties were found among the same demographic groups, although in some cases the patterns differed. For example, the percentage with functional difficulties was significantly higher among adults aged 60–64 years (44.7%) compared with 65–74 years (29.0%) and 75–84 years (32.6%) and among blacks or African Americans (61.6%) compared with whites (29.1%) and Asians/Native Hawaiians or Other Pacific Islanders (16.2%) (Table 1). By state, the percentage reporting increased confusion or memory loss ranged from 6.4% in Tennessee to 20.0% in Arkansas. Among those with increased confusion or memory loss, the percentage with functional difficulties ranged from 21.3% in Wisconsin to 52.2% in West Virginia (Table 2).

Among persons reporting increased confusion or memory loss, those with functional difficulties were significantly more likely than those without functional difficulties to report needing help (81.0% compared with 38.2%), getting help from a family member or friend (46.5% compared with 6.0%), and discussing their increased confusion or memory loss with a health-care provider (32.6% compared with 12.1%). In addition, those who reported functional difficulties were more likely to report being unable to work (32.8% compared with 9.6%) (Table 3).

## Editorial Note

Age is the best-known risk factor for Alzheimer’s disease (the most common cause of dementia), and more than 90% of cases occur in persons aged ≥60 years (2). Research shows that Alzheimer’s disease causes changes in the brain years and even decades before the first symptoms appear, and a better understanding about normal age-related cognitive decline could provide important insights for future prevention efforts (1,2). A systematic review found that among the primary care populations studied, as many as 66% of all dementia cases were undiagnosed, with the majority of missed cases classified as mild to moderate (5). Missed or delayed diagnosis impedes the ability to identify and intervene for treatable causes and to provide timely and accurate information and resources to patients and their families.

Public health surveillance provides the ability to track and monitor trends and identify health disparities to understand the magnitude of the problem, plan for future resource and service needs, inform interventions, and guide research efforts. However, public health surveillance of dementia is limited and complicated by methodologic challenges associated with identifying cases in the community (6). For these reasons, one suggestion is that public health surveillance of these conditions be broadly focused and address outcomes related to functional impairment rather than etiology (6). BRFSS provides an opportunity to respond to the national call for expanded surveillance efforts by tracking self-reported confusion or memory loss that is currently causing functional difficulties among noninstitutionalized adults and could progress to a more serious state of impairment.

The BRFSS results for 21 states described in this report indicate that 12.7% of persons aged ≥60 years report increased confusion or memory loss in the preceding year, and among these persons, 35.2% report functional difficulties. The findings show that increased confusion or memory loss generally increased with age, but the percentage reporting functional difficulties among persons aged 60–64 years was as great as among persons aged ≥85 years and greater than among persons aged 65–84. These findings suggest a need for future studies to examine the relationship of age and functional difficulties caused by increased confusion or memory loss. For example, younger persons might face challenges obtaining diagnostic testing because health-care professionals might not suspect symptoms, or access to employer-sponsored benefits could be placed in jeopardy if employed persons lose their jobs or are unable to work (7)*.*

Among persons reporting functional difficulties, only 32.6% report discussing their symptoms with a health-care provider. Early and accurate diagnosis provides opportunities for individuals and families to initiate financial planning, develop advance directives, enroll in clinical trials and anticipate care needs. Some causes for cognitive decline are reversible (e.g., depression, infections, medication side effects, or nutritional deficiencies), but they can be serious and should be treated by a health-care provider as soon as possible (2). Misperceptions about dementia-related conditions might lead to delayed diagnosis (4), and understanding cultural beliefs and public perception is important for meeting national goals for increasing awareness. For example, studies conducted with diverse groups of older adults found that terminology used to describe brain health and beliefs about cognition varied among racial/ethnic populations (9). Increased confusion or memory loss and functional difficulties were reported among all racial/ethnic groups in this analysis, with persons identifying themselves as black or African American reporting the highest levels of functional difficulties compared with other groups.

What is already known on this topic?

Cognitive decline can negatively affect a person’s life and might progress into a more serious state of impairment or dementia. Memory problems typically are one of the first warning signs of cognitive decline, and up to two thirds of conditions that meet the criteria for dementia are undiagnosed. When diagnosed early and accurately, opportunities exist to treat potentially reversible causes, initiate financial planning, develop advance directives, enroll in clinical trials, and anticipate care needs. National plans call for expanding data and surveillance efforts to track dementia and its impact on individual and population health in the United States.

What is added by this report?

Approximately one in eight adults aged ≥60 years surveyed from 21 states reported increased confusion or memory loss in the preceding year. Among these persons, 35.2% experienced difficulties resulting from confusion or memory loss. Wide variation in these results was found across the 21 states. Respondents who reported functional difficulties were significantly more likely than those who did not to report needing help (81.0% compared with 38.2%), getting help from a family member or friend (46.5% compared with 6.0%), and talking with a health-care provider about their increased confusion or memory loss (32.6% compared with 12.1%).

What are the implications for public health practice?

These findings underscore the need to facilitate discussions with health-care and service providers so that linkages can be made to accurate information and needed services. They also indicate the importance of state-based surveillance to estimate the magnitude of the problem among older adults living in the community.

Among those reporting increased confusion or memory loss and functional difficulties, 81.0% report needing assistance, and only 46.5% report getting help from a family member or friend. The need for care could precede or follow a diagnosis of dementia and escalates over time (8). Care could be provided by family members and friends or through paid services*.* Understanding who is at risk for requiring care now or in the future can help with anticipating needs and associated costs.

Wide variation observed among the 21 states might be the result of different cultural or other factors and indicates the importance of state-based data on this subject. Understanding cultural and social contexts is important when communicating public health messages (8)*.* Future studies of state-specific data examining associations between increased confusion or memory loss and potential risk factors for dementia such as cardiovascular disease, diabetes, depression, or physical inactivity (3) might provide more insights that could also help explain the variations observed across states.

The findings in this report are subject to at least five limitations. First, data are self-reported, not validated by any clinical measurement, and might be subject to recall bias. Second, the survey design is cross-sectional, and causality of specific diseases or conditions cannot be inferred. Third, although questions underwent multiple rounds of cognitive testing to ensure that respondents understood the questions, given misperceptions surrounding dementia (4,7,8)*,* respondents might provide the most “socially acceptable” answer, which could vary by race/ethnicity or geography, and could account in part for the variability observed among states. For example, blacks or African Americans might be less likely than whites to report cognitive decline (10)*.* Furthermore, whether increased confusion or memory loss interferes with a respondent’s ability to accurately describe functional difficulties is unknown. Fourth, these results might underestimate confusion or memory loss and functional difficulties because BRFSS does not include residents of nursing homes or other facilities where a high percentage of people with cognitive impairment reside, and results were limited to landline telephone survey responses and did not include cell phone respondents. Finally, response rates among the 21 states were low and varied widely, ranging from 37.4% to 66.0%.

In May 2012, The U.S. Department of Health and Human Services released the *National Plan to Address Alzheimer’s Disease* (4), which includes a call to strengthen data and surveillance efforts. CDC’s Healthy Brain Initiative is working with the Alzheimer’s Association and numerous other national, state, and local partners to develop a set of public health actions to promote cognitive health as a vital, integral, component of public health and also to address issues related to cognitive impairment for persons living in the community and their care partners (i.e., informal and paid caregivers and health-care providers). This report provides a baseline estimate of the extent of self-reported increased confusion or memory loss and functional difficulties occurring in the preceding year among noninstitutionalized persons aged ≥60 years who might require services and supports now or in the future. The findings underscore the need to facilitate timely discussions with health-care and service providers so that linkages can be made to accurate information and needed services.

## Figures and Tables

**TABLE 1:** Self-reported increased confusion or memory loss (CML) and associated functional difficulties among adults aged ≥60 years, by selected characeristics — Behavioral Risk Factor Surveillance System, 21 states, 2011

Characteristic	Increased CML				Functional difficulties among those with increased CML			
	
	Unweighted no. in sample	Unweighted no. with increased CML	Weighted % reporting increased CML	(95% CI)	Unweighted no. in sample	Unweighted no. with increased CML	Weighted % reporting increased CML	(95% CI)
**21 states overall**	**59,852**	**6,807**	**12.7**	**(12.1–13.3)**	**6,654**	**2,254**	**35.2**	**(32.7–37.8)**
**Age group (yrs)**								
60–64	14,943	1,507	12.0	(10.8–13.2)	1,469	611	44.7	(39.2–50.2)
65–74	24,383	2,505	11.9	(11.0–12.8)	2,444	742	29.0	(25.4–32.9)
75–84	15,718	2,058	14.0	(12.9–15.2)	2,022	618	32.6	(28.0–37.5)
≥85	4,808	737	15.6	(13.7–17.7)	719	283	37.8	(31.1–44.9)
**Sex**								
Men	21,550	2,677	13.4	(12.4–14.4)	2,606	827	34.5	(30.5–38.7)
Women	38,302	4,130	12.1	(11.4–12.9)	4,048	1,427	35.9	(32.8–39.2)
**Race/Ethnicity** *								
White	49,365	5,475	12.1	(11.5–12.7)	5,346	1,615	29.1	(26.7–31.6)
Black or African American	4,697	529	11.8	(9.9–14.0)	522	287	61.6	(52.9–69.6)
Hispanic or Latino	1,621	232	16.9	(13.8–20.6)	229	118	56.2	(45.3–66.5)
Asian/NHOPI	1,536	161	13.8	(8.4–21.9)	160	50	16.2	(4.6–43.8)
AI/AN	465	73	13.7	(7.8–22.9)	71	38	45.2	(21.6–71.2)
Other race/Multiracial	1,388	218	15.3	(11.2–20.4)	215	86	39.4	(26.1–54.5)
**Education level**								
Less than high school diploma	6,791	1,019	16.2	(14.4–18.2)	988	522	52.9	(46.5–59.3)
High school diploma	19,580	2,234	12.5	(11.5–13.6)	2,194	756	35.3	(30.6–40.3)
Some college	15,279	1,738	12.1	(11.2–13.2)	1,702	524	28.3	(24.5–32.5)
College graduate	18,077	1,803	10.9	(9.9–12.0)	1,757	446	24.2	(20.0–28.9)
**Disability status**								
Disabled	24,339	4,363	20.2	(19.1–21.3)	4,263	1795	44.4	(41.1–47.7)
Not disabled	35,254	2,410	7.5	(6.9–8.1)	2,357	451	18.3	(15.3–21.7)
**Veteran status**								
Veteran	12,061	1,610	13.9	(12.6–15.2)	1,566	480	34.4	(29.1–40.2)
Not a veteran	47,764	5,193	12.3	(11.7–13.0)	5,085	1,773	35.5	(32.7–38.5)
**Employment status**								
Employed/Self-employed	12,447	920	7.8	(6.9–8.9)	899	174	24.4	(18.7–31.2)
Unemployed	1,426	200	16.4	(12.7–20.9)	196	87	49.1	(35.5–62.8)
Homemaker	4,097	457	11.8	(10.0–14.0)	453	141	34.5	(26.1–43.9)
Student	61	7	3.9	(1.5–10.0)	6	2	—	—
Retired	37,781	4,198	12.3	(11.5–13.0)	4,104	1,219	27.7	(24.7–30.8)
Unable to work	3,846	989	28.3	(25.1–31.7)	961	612	65.0	(58.5–71.0)

**Abbreviations:** CI = confidence interval; NHOPI = Native Hawaiian or Other Pacific Islander; AI/AN = American Indian/Alaska Native.

* Race/ethnicity was coded into six mutually exclusive categories: white, black or African American, Hispanic or Latino, Asian/NHOPI, AI/AN, and other race/multiracial. Persons who self-identified as Hispanic might be of any race. Persons who self-identified as any of the other five categories were all non-Hispanic.

**TABLE 2:** Self-reported increased confusion or memory loss (CML) and associated functional difficulties among adults aged ≥60 years, by state — Behavioral Risk Factor Surveillance System, 21 states, 2011

	Increased CML			Functional difficulties among those with increased CML				
		
State	Unweighted no. in sample	Unweighted no. with increased CML	Weighted % reporting increased CML	(95% CI)	Unweighted no. in sample	Unweighted no. with increased CML	Weighted % reporting associated difficulties	(95% CI)
**21 states overall**	**59,852**	**6,807**	**12.7**	**(12.1–13.3)**	**6,654**	**2,254**	**35.2**	**(32.7–37.8)**
Arkansas	2,127	374	20.0	(17.9–22.3)	371	135	38.6	(32.5–45.0)
California	2,073	328	17.0	(14.9–19.3)	328	95	30.0	(23.9–36.9)
Florida	5,194	651	13.8	(12.2–15.7)	637	232	42.0	(34.7–49.8)
Hawaii	3,108	335	9.2	(8.0–10.6)	333	115	38.4	(31.2–46.2)
Illinois	2,193	241	11.4	(9.7–13.4)	241	80	39.1	(30.6–48.3)
Iowa	2,827	233	9.0	(7.8–10.4)	232	62	31.1	(23.8–39.4)
Louisiana	4,424	303	7.3	(6.2–8.5)	297	122	43.4	(35.4–51.8)
Maryland	1,805	168	9.5	(7.6–11.7)	165	40	24.7	(16.6–35.0)
Michigan	1,461	208	13.9	(11.4–16.9)	208	57	31.2	(21.6–42.8)
Nebraska	4,705	578	12.0	(10.8–13.4)	576	211	33.3	(28.3–38.7)
New Hampshire	2,447	262	11.0	(9.6–12.6)	183	58	33.6	(26.1–42.1)
New York	1,232	131	10.6	(8.6–13.0)	129	42	39.5	(29.1–51.0)
North Carolina	4,618	393	8.5	(7.3–9.8)	385	153	43.3	(35.7–51.3)
Oklahoma	1,810	212	12.1	(10.5–14.0)	210	70	35.7	(28.3–43.8)
South Carolina	5,062	610	13.7	(12.1–15.4)	598	248	39.7	(33.3–46.4)
Tennessee	2,586	159	6.4	(5.2–7.7)	148	68	47.1	(36.7–57.7)
Texas	2,922	394	12.6	(10.8–14.6)	391	138	37.8	(30.3–45.9)
Utah	973	166	17.0	(14.4–19.9)	164	42	30.2	(22.2–39.6)
Washington	4,360	697	15.7	(14.4–17.1)	695	154	22.3	(18.5–26.5)
West Virginia	2,061	156	8.3	(7.0–9.9)	155	78	52.2	(43.1–61.2)
Wisconsin	1,864	208	11.1	(9.0–13.5)	208	54	21.3	(14.8–29.6)

**Abbreviation:** CI = confidence interval.

**TABLE 3:** Selected characteristics of adults aged ≥60 years with self-reported increased confusion or memory loss (CML), with and without associated functional difficulties — Behavioral Risk Factor Surveillance System, 21 states, 2011

	Those with self-reported increased CML		Those with CML and any functional difficulty		Those with CML and without functional difficulty		
				
Characteristic	%	(95% CI)	%	(95% CI)	%	(95% CI)	p value
**Total no. of respondents per category**	**6,654**		**2,254**		**4,400**		
Needs help*	53.1	(50.5–55.7)	81.0	(77.3–84.3)	38.2	(35.3–41.2)	<0.001
Always, usually or sometimes receives help from family member or friend	20.1	(17.9–22.5)	46.5	(41.8–51.3)	6.0	(4.6–7.6)	<0.001
Discussed increased CML with health-care provider	19.3	(17.3–21.4)	32.6	(28.3–37.3)	12.1	(10.3–14.1)	<0.001
Unable to work	17.8	(15.7 – 20.0)	32.8	(28.4 – 37.5)	9.6	(7.8 – 11.7)	<0.001
Lives alone	34.6	(32.4–36.8)	38.7	(34.6–42.9)	32.4	(29.9–35.0)	0.011

**Abbreviation:** CI = confidence interval.

* Respondents indicated that they needed help in one of the following areas as a result of their increased confusion or memory loss: safety, transportation, household activities, personal care, or needs assistance in some other area.

## Footnotes

* Additional information, including complete survey questions, available at http://www.cdc.gov/brfss.

† Arkansas, California, Florida, Hawaii, Illinois, Iowa, Louisiana, Maryland, Michigan, Nebraska, New Hampshire, New York, North Carolina, Oklahoma, South Carolina, Tennessee, Texas, Utah, Washington, West Virginia, and Wisconsin.

§ Additional information available at http://www.cdc.gov/aging/healthybrain/brfss-faq.htm.

¶ Excluded were 2.8% of otherwise eligible participants from the seven states.

** Response rates for BRFSS are calculated using standards set by American Association of Public Opinion Research response rate formula no. 4, available at http://www.aapor.org/standard_definitions2.htm. The response rate is the number of respondents who completed the survey as a proportion of all eligible and likely eligible persons.

†† Race/ethnicity was coded into six mutually exclusive categories: white, black or African American, Hispanic or Latino, Asian/Native Hawaiian or Pacific Islander, American Indian/Alaska Native, and other race/multiracial. Persons who self-identified as Hispanic might be of any race. Persons who self-identified as any of the other five categories were non-Hispanic.

§§ Respondents indicated limitation in any way in activities because of physical, mental, or emotional problems, or indicated use of special equipment such as a cane or wheelchair.
